# A novel autophagy inhibitor berbamine blocks SNARE-mediated autophagosome-lysosome fusion through upregulation of BNIP3

**DOI:** 10.1038/s41419-018-0276-8

**Published:** 2018-02-14

**Authors:** Ruoqiu Fu, Qin Deng, Hongwei Zhang, Xiaoye Hu, Yunong Li, Yanxia Liu, Jinjiao Hu, Qingsong Luo, Yanhao Zhang, Xiuxing Jiang, Lirong Li, Chong Yang, Ning Gao

**Affiliations:** College of Pharmacy, Third Military Medical University, Chongqing, 400038 China

## Abstract

Increasing evidences reveal that autophagy inhibitor could enhance the effect of chemotherapy to cancer. However, few autophagy inhibitors are currently approved for clinical application in humans. Berbamine (BBM) is a natural compound extracted from traditional Chinese medicine that is widely used for treatment of a variety of diseases without any obvious side effects. Here we found that BBM is a novel auophagy inhibitor, which potently induced the accumulation of autophagosomes by inhibiting autophagosome-lysosome fusion in human breast cancer cells. Mechanistically, we found that BBM blocked autophagosome-lysosome fusion by inhibiting the interaction of SNAP29 and VAMP8. Furthermore, BBM induced upregulation of BNIP3 and the interaction between SNAP29 and BNIP3. BNIP3 depletion or SNAP29 overexpression abrogated BBM-mediated blockade of autophagosome-lysosome fusion through the interaction between SNAP29 and VAMP8, whereas BNIP3 overexpression blocked autophagosome-lysosome fusion through inhibition of the interaction between SNAP29 and VAMP8. These findings suggest that upregulation of BNIP3 and interaction between BNIP3 and SNAP29 could be involved in BBM-mediated blockade of autophagosome-lysosome fusion through inhibition of the interaction between SNAP29 and VAMP8. Our findings identify the critical role of BNIP3 in blockade of autophagosome-lysosome fusion mediated by BBM, and suggest that BBM could potentially be further developed as a novel autophagy inhibitor, which could enhance the effect of chemotherapy to cancer.

## Introduction

Autophagy is an important homeostatic cellular recycling mechanism responsible for degrading unnecessary or dysfunctional cellular organelles and proteins in all living cells^1^. Initially, parts of the cytoplasm and cellular organelles are engulfed within a double-membrane vesicle called the autophagosome. The autophagosome fuses with lysosomes to form an autolysosome, which results in the degradation of the sequestered materials by various lysosomal hydrolytic enzymes^2^. Specific membrane fusion is generally achieved by soluble *N*-ethylmaleimide-sensitive factor attachment protein receptor (SNARE) complexes^3^. It has recently been reported that an autophagosomal SNARE syntaxin 17 (STX17) interacts with cytosolic SNARE SNAP29 and the lysosomal SNARE VAMP8, and all of these proteins are required for autophagosome-lysosome fusion^4^. However, the molecular mechanism for how the fusion of completed autophagosomes with the lysosome is regulated has not been fully understood.

BNIP3 is a protein with homology to BCL2 in its BH3 domain^5,6^, and can be expressed upon hypoxia and drive mitophagy in many different cell types^7–9^. It has been shown recently that BNIP3 may play an important role in the regulation of autophagosome-lysosome fusion^10^. However, the mechanistic insight discriminating the function of BNIP3 in the regulation of autophagosome-lysosome fusion remains unclear.

Berbamine (BBM), a natural bisbenzyl isoquinoline alkaloid, is isolated from the traditional Chinese medicine *Berberis amurensis Rupr*. BBM has been used to treat patients with a low level of white blood cells caused by chemotherapy and/or radiotherapy^11^. BBM has been reported to have antitumor activities in various types of cancers, including myeloma, lung, and breast cancers^12–14^. More recently, only one report showed that BBM postcondition protects the heart from ischemia/reperfusion injury through modulation of autophagy^15^. However, the detailed mechanism by which BBM regulates autophagy in human breast cancer cells remains unclear.

In the present study, we found for the first time that BBM potently induced the accumulation of autophagosome through inhibiting autophagosome-lysosome fusion. Mechanistic study revealed that BBM blocked autophagosome-lysosome fusion by inhibiting the interaction of SNAP29 and VAMP8. Furthermore, BBM induced upregulation of BNIP3, which interacts with SNAP29, resulting in inhibition of the interaction between SNAP29 and VAMP8, leading, in turn, to blockade of autophagosome-lysosome fusion, and culminating in accumulation of autophagosome.

Because BBM exhibits low toxicity^16^, development to exploit BBM that can efficiently modulate autophagy either alone or in combination with other chemotherapy may represent a novel therapeutic strategy for treatment of breast cancer.

## Results

### **BBM enhances LC3B**-**II stability and puncta formation in multiple cancer cells**

**Fig.1:**
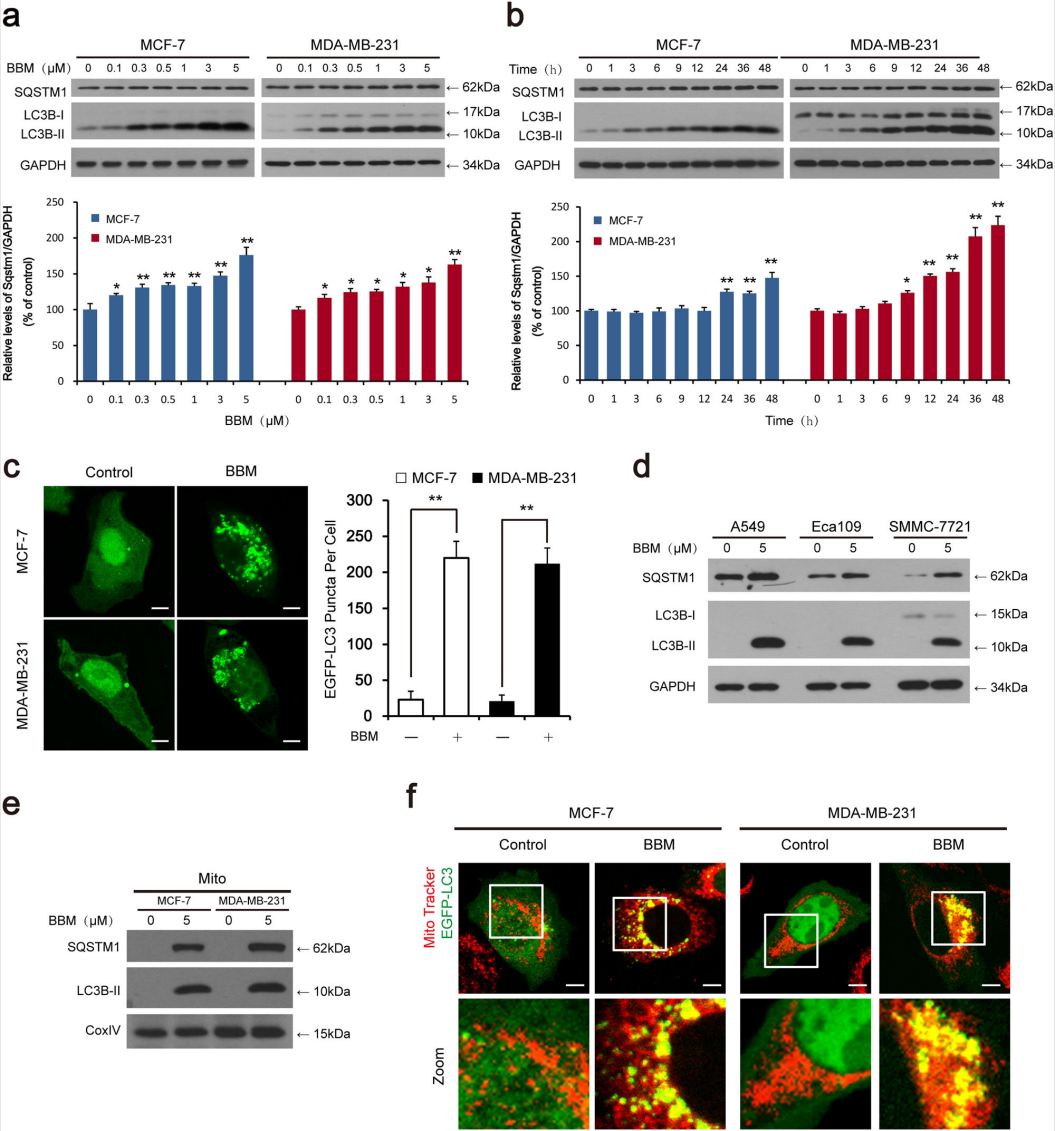
BBM induces autophagic alterations in MCF-7 and MDA-MB-231 cells. **a**, **b** Cells were exposed to various concentrations of BBM for 24 h, or treated with BBM (5 μM) for different time intervals as indicated. The expression of autophagy-related proteins (LC3B-II and SQSTM1) was detected by western blot. The relative levels of SQSTM1 were quantified by densitometry and normalized to GAPDH in three independent experiments (mean ± SD, *P < 0.05, **P < 0.01 compared with control). **c** Cells expressed with EGFP-LC3 were treated with BBM (5 μM) for 24 h, the EGFP-LC3 puncta was observed under confocal microscopy; scale bars: 10 μm. The EGFP puncta per cell was quantified from 30 cells in three independent experiments; data were presented as mean ± SD (**P < 0.01). **d** Various cancer cells (including human lung adenocarcinoma cell A549, human esophageal cancer cell Eca109, and human hepatocellular carcinoma cell SMMC-7721) were treated with BBM (5 μM) for 24 h. The expression of autophagy-related proteins (LC3B-II and SQSTM1) was detected by western blot. **e** Cells were treated with BBM (5 μM) for 24 h. The mitochondrial fractions were prepared, and then the expressions of LC3B-II and SQSTM1 in mitochondrial fractions (Mito) were detected by western blot analysis. The COXIV was used as a loading control. **f** Cells expressed with EGFP-LC3 were treated with BBM (5 μM) for 24 h. The colocalization of EGFP-LC3 and MitoTracker (Deep Red FM) was examined by confocal microscopy. Scale bars: 10 μm

We first examined the expression of LC3B-II and SQSTM1 in both MCF-7 and MDA-MB-231 cells treated with BBM by using western blot analysis. Treating cells with BBM resulted in dose- and time-dependent accumulation of LC3B-II in both cell lines (Fig. 1a, b). We also used MCF-7 and MDA-MB-231 cells transiently expressing EGFP-LC3 to determine autophagosome accumulation by confocal laser-scanning microscope. Treating cells with BBM resulted in a marked increase in EGFP-LC3 puncta formation in MCF-7 and MDA-MB-231 cells (Fig. 1c). To determine whether BBM-mediated autophagy observed in breast cancer cells also occur in other cancer cell lines, parallel studies were carried out in A549 (human lung adenocarcinoma cell line), Eca109 (human esophageal cancer cell line), and SMMC-7721 (human hepatocellular carcinoma cell line) cells. Similarly, BBM treatment caused the marked accumulation of LC3B-II and SQSTM1 in these cells (Fig. 1d). To examined whether BBM affects the mitophagy, the expression of LC3B-II and SQSTM1 in mitochondrial fractions were determined by western blot analysis. Treatment of cells with BBM resulted in accumulation of LC3B-II and SQSTM1 in the mitochondria of both MCF-7 and MDA-MB-231 cells (Fig. 1e). The immunofluorescence analysis showed that the colocalization of EGFP-LC3 puncta and MitoTracker (Deep Red FM) was observed in cells treated with BBM (Fig. 1f). These results indicated that BBM may influence the process of mitophagy in MCF-7 and MDA-MB-231 cells.

### **BBM inhibits autophagic flux in breast cancer cells**

**Fig. 2:**
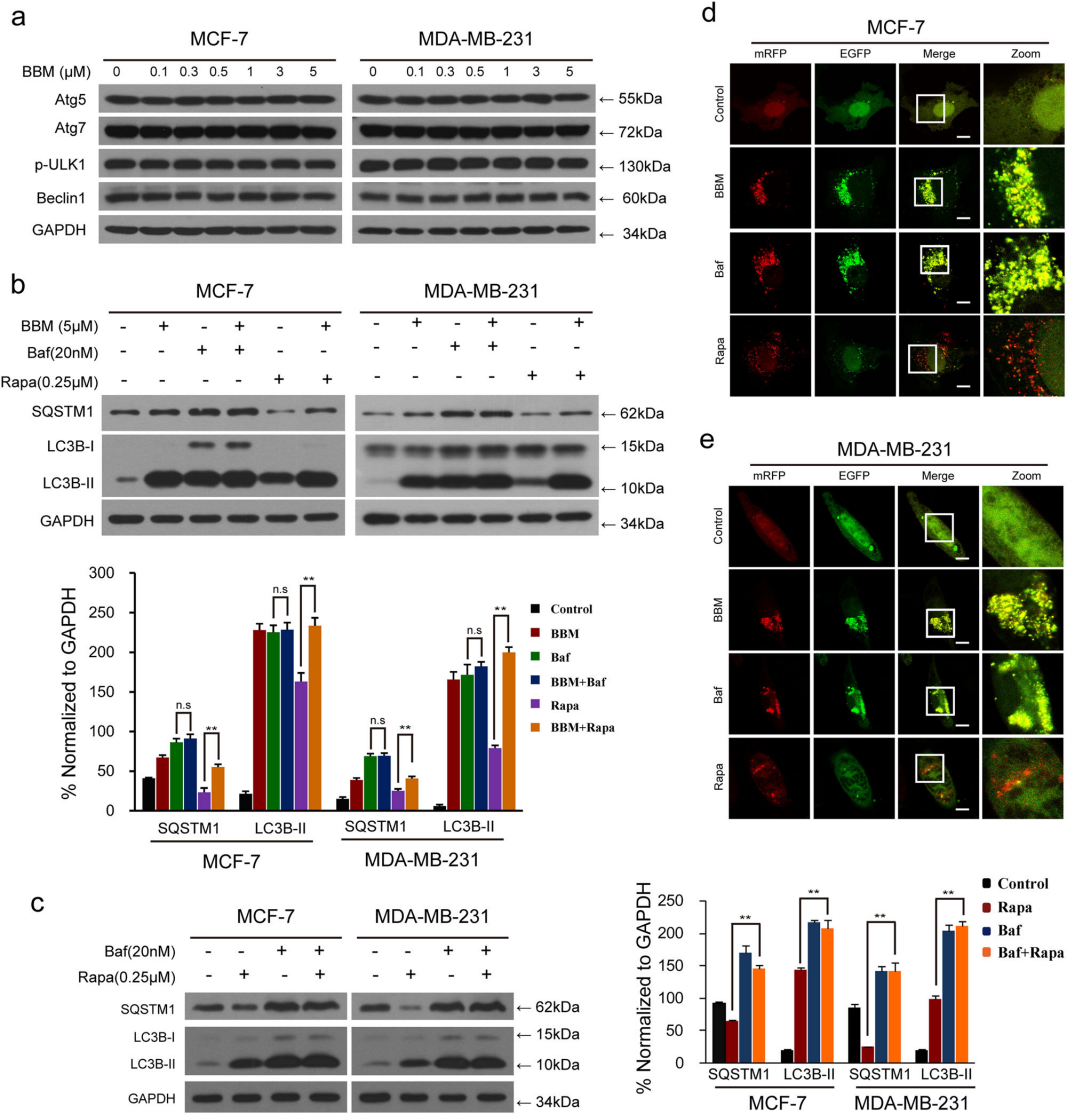
BBM inhibits autophagic degradation in MCF-7 and MDA-MB-231 cells. **a** Cells were exposed to various concentrations of BBM for 24 h. The expression of autophagy-related proteins (Atg5, Atg7, p-ULK1, and Beclin1) was detected by western blot. **b** Cells were treated without or with BBM (5 μM) in the presence or absence of 20 nM bafilomycin A1 (Baf) or 0.25 μM rapamycin (Rapa) for 24 h; the expression of SQSTM1 and LC3B-II was analyzed by western blot. Comparisons of the intensities were statistically estimated and represented as mean ± SD for three independent experiments (ns, not significant; **P < 0.01). **c** Cells were treated with Rapa (0.25 μM) in the presence or absence of Baf (20 nM) for 24 h; the expression of SQSTM1 and LC3B-II was analyzed by western blot. Comparisons of the intensities were statistically estimated and represented as mean ± SD for three independent experiments (ns, not significant; **P < 0.01). **e**, **f** Cells were transfected with a tandem fluorescent LC3 (tfLC3), and were exposed to BBM (5 µM), Baf (20 nM), and Rapa (0.25 μM), respectively, for 24 h. The colocalization of mRFP and EGFP-LC3 puncta was examined by confocal microscopy. Scale bars: 10 μm

Autophagy is a highly regulated process, in which the activities of autophagy-related (ATG) proteins are involved^17^. Next, we determined whether BBM affects the activities of autophagy-related proteins in breast cancer cells. Unfortunately, the autophagy-related proteins such as p-ULK1, ATG5, ATG7, and Beclin1 were not changed in cells treated with BBM (Fig. 2a), suggesting that BBM treatment does not affect autophagic vesicle nucleation and autophagosome formation.

Our earlier observations showed that treatment with BBM increased the levels of SQSTM1 in multiple cancer cells (Fig. 1a, b, d), suggesting that BBM may inhibit autophagic degradation^18,19^. To further confirm these observations, we examined the effects of BBM on accumulation of LC3B-II and SQSTM1 in the presence or absence of bafilomycin A1 (Baf) or rapamycin (Rapa) by using western blot analysis. As shown in Fig. 2b, BBM treatment resulted in accumulation of LC3B-II and SQSTM1, which was similar to the result caused by Baf. Compared with BBM or Baf treatment alone, combined treatment with BBM and Baf did not show any significant increases in accumulation of LC3B-II and SQSTM1. In contrast, treatment with Rapa, which is known to induce autophagy, resulted in a slight increase in levels of LC3B-II that were further enhanced by BBM or Baf (Fig. 2b, c). Furthermore, treatment with Rapa led to decreased SQSTM1 levels that were markedly reversed by BBM or Baf (Fig. 2b, c).

In order to further understand the inhibitory effects of BBM on autophagic flux, cells transfected with a tandem reporter construct (tandem fluorescent LC3; tfLC3) were treated with BBM (5 µM, 24 h) followed by assessment of EGFP-LC3 and mRFP-LC3 puncta colocalization. Treatment with BBM or Baf caused pronounced formation of LC3 puncta that displayed both green and red fluorescence intensity producing a yellow overlay (Fig. 2d, e). In contrast, cells exposed to Rapa led to the production of large amounts of red-only puncta (Fig. 2d, e). These findings suggest that BBM inhibits the late stage of autophagy, thereby resulting in a marked accumulation of autophagosomes.

### BBM inhibits autolysosome formation by interfering with the fusion of autophagosome with lysosome

**Fig. 3:**
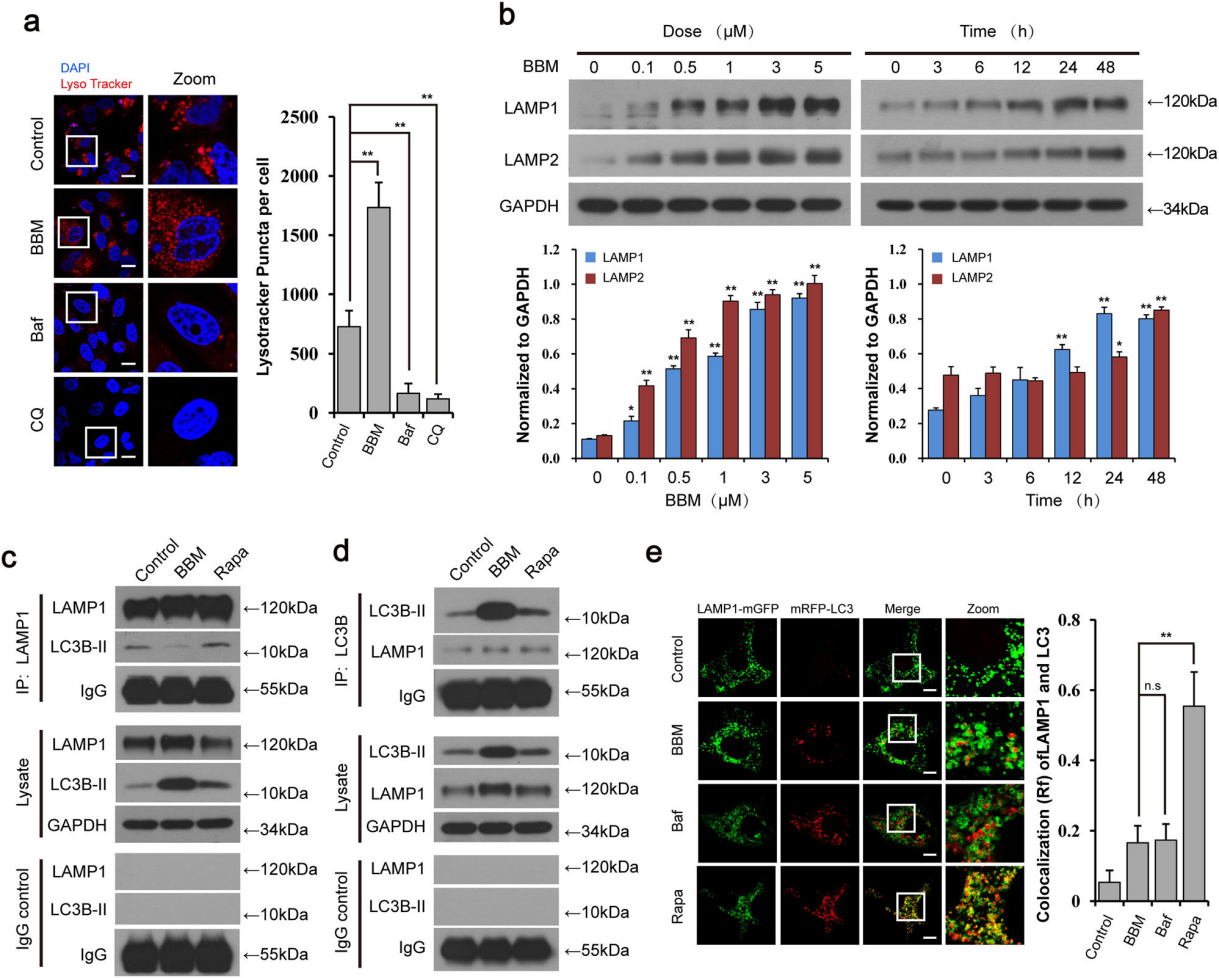
BBM blocks autophagosome-lysosome fusion but does not alter the lysosome pH. **a** MCF-7 cells were treated with BBM (5 µM), Baf (20 nM), or CQ (20 μM) for 24 h. The fluorescent signals were detected by confocal microscopy after stained with LysoTracker Red. Scale bars: 25 µm. The average LysoTracker puncta per cell was quantified from 30 cells in three independent experiments (mean ± SD, **P < 0.01). **b** MCF-7 cells were exposed to various concentrations of BBM for 24 h, or treated with BBM (5 μM) for different time intervals as indicated. The expression of LAMP1 and LAMP2 was determined by western blot. The relative levels of LAMP1 and LAMP2 were quantified by densitometry and normalized to GAPDH in three independent experiments (mean ± SD, *P < 0.05, **P < 0.01 compared with control). **c**, **d** MCF-7 cells were treated with BBM (5 µM) or Rapa (0.25 µM) for 24 h, whole-cell lysate was prepared and subjected to immunoprecipitation using anti-LAMP1 (left panel) or anti-LC3B (right panel), and the associated LC3B-II and LAMP1 were determined using immunoblotting. **e** MCF-7 cells co-transfected with LAMP1-mGFP and mRFP-LC3 were treated with vehicle, BBM (5 µM), Baf (20 nM), or Rapa (0.25 μM) for 24 h, the colocalization of LAMP1-mGFP and mRFP-LC3 puncta was examined by confocal microscopy. The Pearson’s correlation coefficient (*R*^2^) of LAMP1-mGFP and mRFP-LC3 colocalization was represented as mean ± SD (ns, not significant; **P < 0.01), 30 cells. Scale bars: 10 μm

The data shown earlier suggest that BBM inhibits the autophagic flux at the late stage of autophagy. Therefore, we attempted to determine the effect of BBM on lysosomal function. The intra-lysosomal pH is a critical factor in determining lysosomal functions^20^. We thus examined the effect of BBM on intra-lysosomal pH by using the LysoTracker Red, a deep red-fluorescent dye for labeling and tracking acidic organelles^21^. As shown in Fig. 3a, BBM treatment did not affect intra-lysosomal pH compared to the control, whereas treatment with Baf or chloroquine (CQ) effectively abolished the fluorescence, suggesting that BBM does not affect the lysosomal acidification and may inhibit autolysosome formation through the different mechanism from that mediated by Baf or CQ.

Lysosome-associated membrane protein-1 (LAMP1) and LAMP2 are major protein components of the lysosomal membrane and play critical roles in autophagosome-lysosome fusion^22^. We next examined the effect of BBM on the expression of LAMP1 and LAMP2. By using immunoblotting, we observed the obvious increases in LAMP1 and LAMP2 protein levels in MCF-7 cells treated with BBM in dose- and time-dependent manners (Fig. 3b), suggesting that the mechanism by which BBM blocked autophagosome-lysosome fusion was not due to reduced expression of LAMP1 and LAMP2.

To further understand the inhibitory effect of BBM on autophagosome-lysosome fusion, immunoprecipitation analysis was employed. As shown in Fig. 3c, d, treatment with BBM resulted in decrease in the interaction of LAMP1 with LC3B-II. We also examined the autophagosome-lysosome fusion process by tracking the late endosome/lysosome marker LAMP1 to detect its colocalization with the autophagosomal marker mRFP-LC3 in mRFP-LC3-expressing MCF-7 cells. As shown in Fig. 3e, mRFP-LC3 was not colocalized with LAMP1-mGFP in cells treated with BBM or Baf. In contrast, there was extensive colocalization mRFP-LC3 and LAMP1-mGFP in cells treated with Rapa. Such observations were confirmed by a quantification analysis for the colocalization coefficient as presented in Fig. 3e. These findings present clear evidence that BBM inhibits autolysosome formation not by affecting the lysosomal function, but by impairing autophagosome-lysosome fusion.

### BBM blocks autophagosome-lysosome fusion by inhibiting the interaction of SNAP29 and VAMP8

**Fig. 4:**
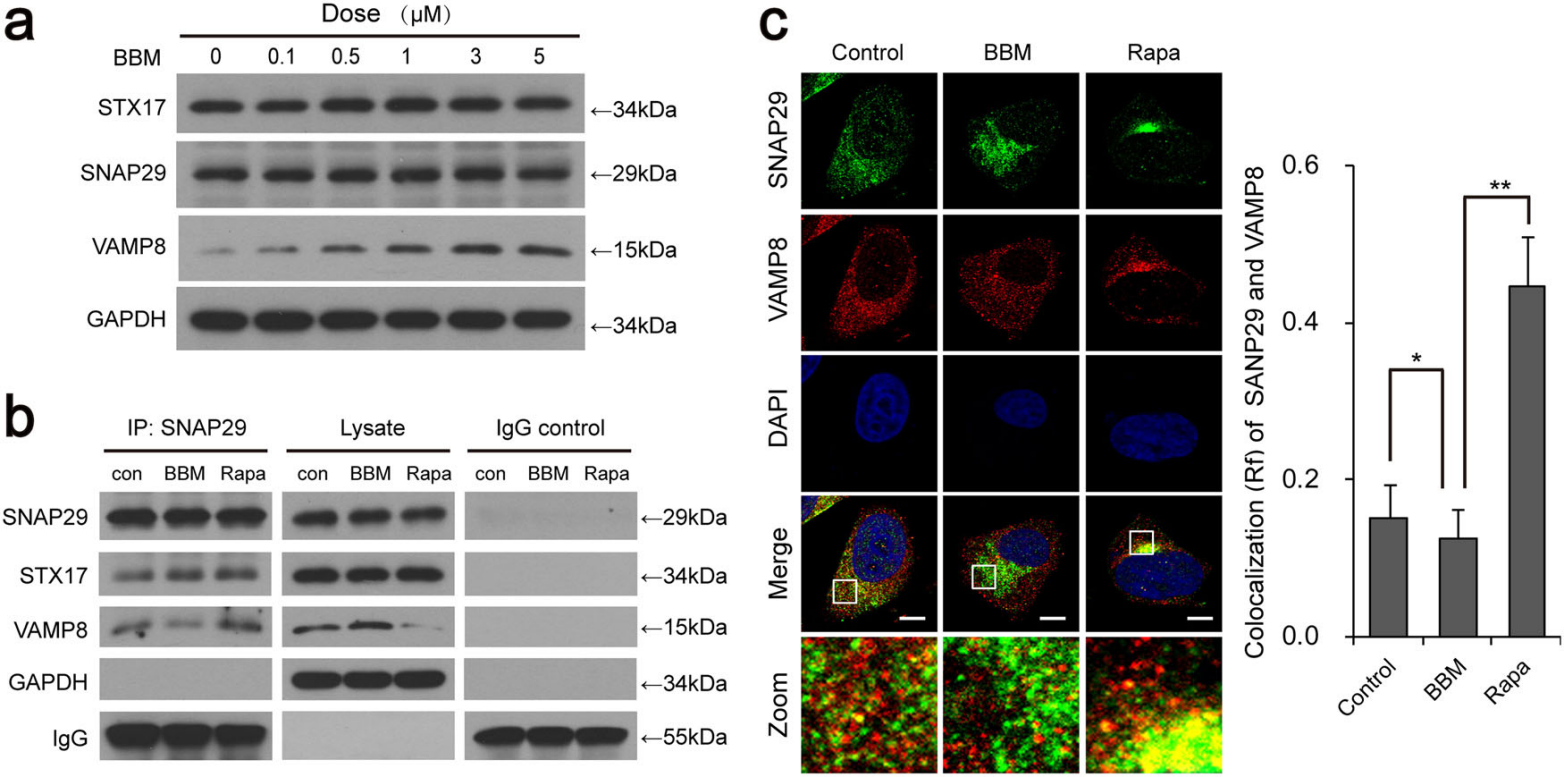
BBM blocks the formation of STX17–SNAP29–VAMP8 complex by inhibiting the interaction of SNAP29 and VAMP8. **a** MCF-7 cells were treated with various concentrations of BBM for 24 h; the expression of STX17, SANP29, and VAMP8 was determined by western blot. **b** MCF-7 cells were treated with BBM (5 µM) or Rapa (0.25 µM) for 24 h, whole-cell lysate was prepared and subjected to immunoprecipitation using anti-SNAP29, and the associated SNAP29, STX17, and VAMP8 were determined using immunoblotting. **c** Confocal microscopy images of MCF-7 cells immunostained for SNAP29 (green) and VAMP8 (red) after treating with BBM (5 μM) or Rapa (0.25 µM) for 24 h. The Pearson’s correlation coefficient (*R*^2^) of SNAP29 and VAMP8 colocalization was represented as mean ± SD (*P < 0.01; **P < 0.01), 30 cells. Scale bars: 10 μm

A number of evidences revealed that the SNAREs are likely to be involved in autophagosome-lysosome fusion^23^. It has been reported that STX17, an autophagosomal SNARE, interacts with SNAP29 and the lysosomal SNARE VAMP8, and all of these proteins are required for autophagosome-lysosome fusion^4^. To further understand the molecular mechanism of BBM-inhibited autophagosome-lysosome fusion, we next examined the effect of BBM on the expression of STX17, SNAP29, and VAMP8 by using western blot analysis. As shown in Fig. 4a, treating cells with BBM resulted in increases in the level of VAMP8 in a dose-dependent manner, whereas BBM did not affect the expression of STX17 and SNAP29, indicating that the mechanism by which BBM blocked autophagosome-lysosome fusion was not due to reduced expression of these proteins.

To determine whether BBM affects the interaction of SNAP29 with either STX17 or VAMP8, immunoprecipitation analysis was employed. As shown in Fig. 4b, immunoprecipitation with an anti-SNAP29 in cell lysates revealed that SNAP29 was co-precipitated with both STX17 and VAMP8 in cell lysates of control cells. However, BBM treatment obviously reduced the co-precipitation of SNAP29 with VAMP8. Treatment with Rapa, an autophagy inducer, increased the interaction of SNAP29 with VAMP8. Similarly, immunofluorescence analysis revealed that the colocalization of SNAP29 and VAMP8 was decreased by BBM treatment, but increased by Rapa treatment (Fig. 4c). Such findings suggest that the BBM-mediated blockade of autophagosome-lysosome fusion was due to impaired recruitment of SNAP29 to lysosomes but not autophagosomes.

### BBM induces upregulation of BNIP3 and the interaction of SNAP29 and BNIP3

**Fig. 5:**
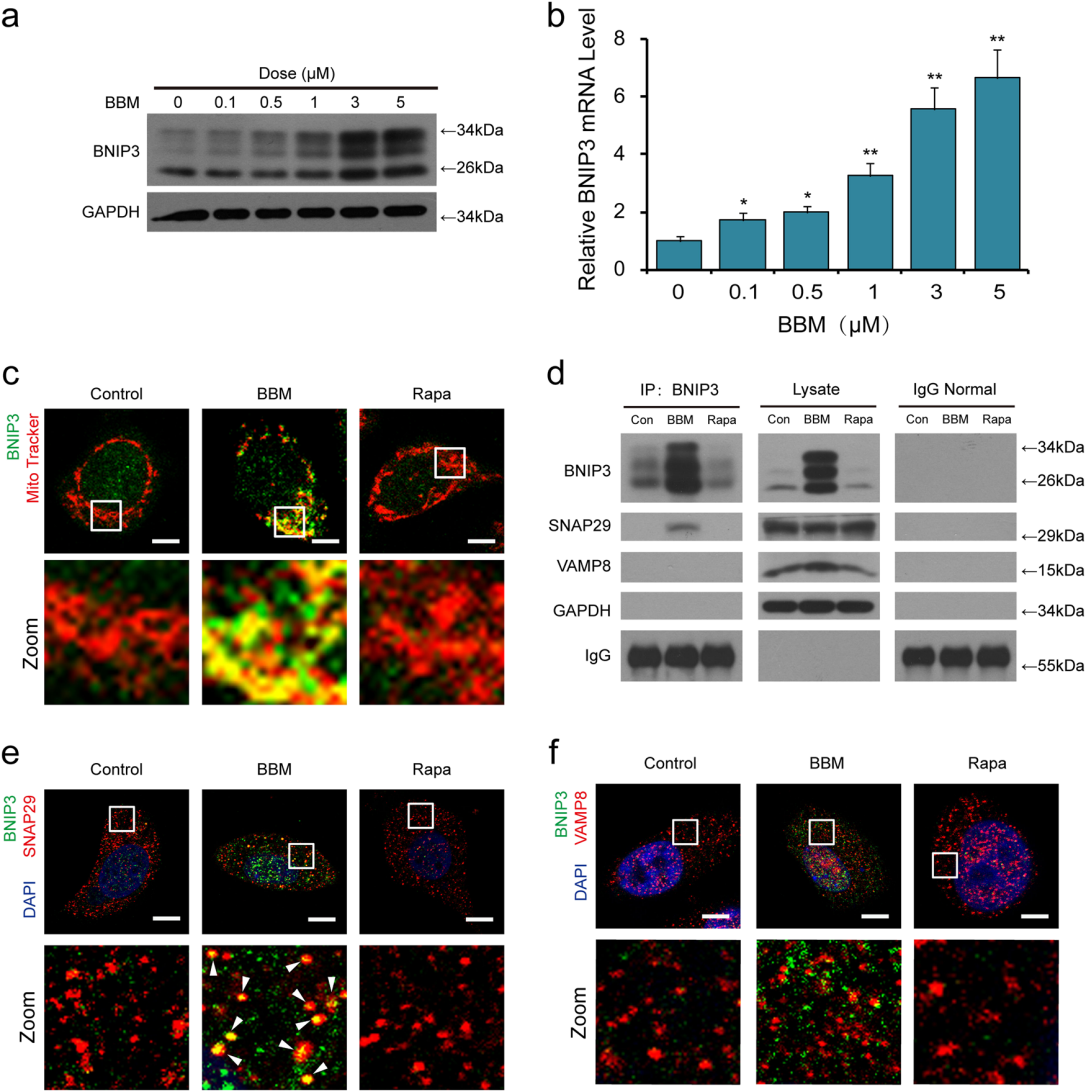
BBM induces upregulation of BNIP3 and the interaction of SNAP29 and BNIP3. MCF-7 cells were treated with various concentrations of BBM for 24 h. **a** The expression of BNIP3 was determined by western blot. **b** The mRNA levels of BNIP3 were determined by qRT-PCR in three independent experiments (mean ± SD, *P < 0.05, **P < 0.01 compared with control). **c** MCF-7 cells were treated with BBM (5 μM) or Rapa (0.25 μM) for 24 h. After immunostaining with BNIP3 (green), the colocalization of MitoTracker (Deep Red FM, red) and BNIP3 was examined by confocal microscopy. Scale bars: 10 μm. **d** MCF-7 cells were treated with BBM (5 µM) or Rapa (0.25 µM) for 24 h, whole-cell lysate was prepared and subjected to immunoprecipitation using anti-BNIP3, and the associated SNAP29 and VAMP8 were determined using immunoblotting. **e** Confocal microscopy images of MCF-7 cells immunostained for BNIP3 (green) and SNAP29 (red) after treatment with BBM (5 μM) or Rapa (0.25 µM) for 24 h. The colocalization puncta was indicated by arrowheads. Scale bars: 10 μm. **f** Confocal microscopy images of MCF-7 cells immunostained for BNIP3 (green) and VAMP8 (red) after treatment with BBM (5 μM) or Rapa (0.25 µM) for 24 h. Scale bars:10 μm

BNIP3 is a mitophagy receptor that is expressed upon hypoxia and drives autophagy^7–9^. Our previous results suggested that BBM may affect the process of mitophagy. Therefore, we examined the effect of BBM on the expression and mRNA levels of BNIP3. As shown in Fig. 5a, b, treating cells with BBM resulted in increasing the expression and the mRNA levels of BNIP3 in a dose-dependent manner. It has been reported that BNIP3 was colocalized with mitochondria^24^. We next examined whether BNIP3 is colocalized with mitochondria in response to BBM treatment. Our results showed that BNIP3 was colocalized with mitochondria in cells treated with BBM (Fig. 5c).

A recent study revealed that BNIP3 expression led to accumulation of autophagosome but not autolysosomes^10^. Such an effect of BNIP3 expression was found to be similar to that of BBM. We then suggesting that BNIP3 may be involved in blockade of autophagosome-lysosome fusion mediated by BBM. To determine whether BNIP3 contributes to blockade of autophagosome-lysosome fusion mediated by BBM, the interaction of BNIP3 with either SNAP29 or VAMP8 in cells treated with BBM was investigated by using immunoprecipitation analysis. We found that BNIP3 was co-precipitated with SNAP29 but not with VAMP8 in cells treated with BBM, whereas BNIP3 was not co-precipitated with either SNAP29 or VAMP8 in cells treated with Rapa (Fig. 5d). Immunofluorescence analysis also revealed that BBM but not Rapa treatment led to colocalization of BNIP3 and SNAP29 (Fig. 5e). However, BNIP3 was not colocalized with VAMP8 in cells treated with either BBM or Rapa (Fig. 5f). Together, these findings suggest that upregulation of BNIP3 and interaction between BNIP3 and SNAP29 could be involved in BBM-mediated blockade of autophagosome-lysosome fusion through inhibition of the interaction between SNAP29 and VAMP8.

### BNIP3 depletion abrogates BBM-mediated blockade of autophagosome-lysosome fusion through the interaction between SNAP29 and VAMP8

**Fig. 6:**
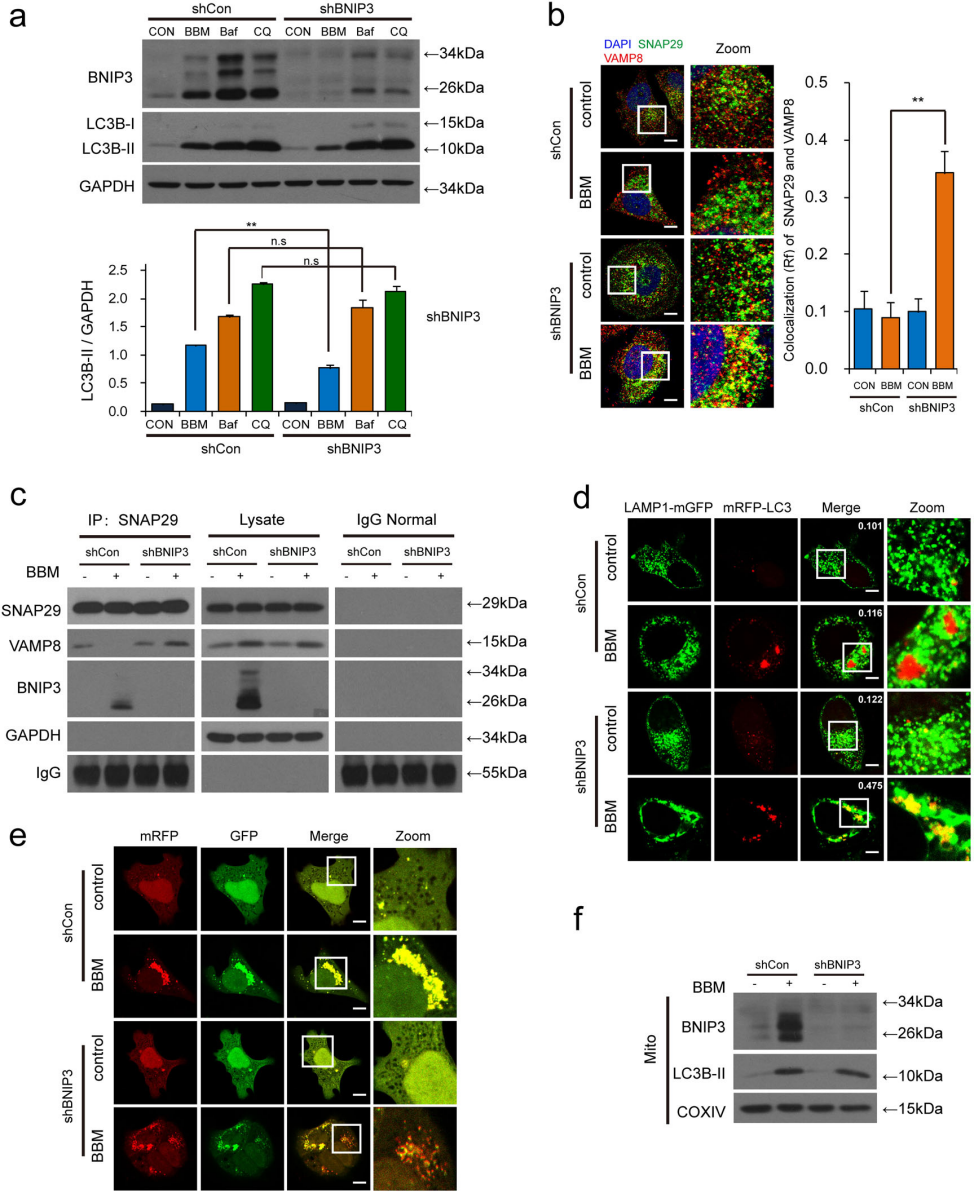
Knocking down BNIP3 attenuates BBM-mediated blockade of autophagosome-lysosome fusion. **a** MCF-7 cells transfected with control shRNA (shCon) or *BNIP3* shRNA (shBNIP3) were treated with BBM (5 μM), Baf (20 nM), or CQ (20 μM) for 24 h; the expression of BNIP3 and LC3B-II was determined by western blot. Comparison of the intensities was statistically estimated and represented as mean ± SD for three independent experiments (ns, not significant, **P < 0.01). **b** Confocal microscopy images of MCF-7 cells immunostained for SNAP29 (green) and VAMP8 (red) after transfected with control shRNA (shCon) or *BNIP3* shRNA (shBNIP3) for 24 h and treatment with BBM (5 μM) for additional 24 h. The Pearson’s correlation coefficient (*R*^2^) of SNAP29 and VAMP8 colocalization was represented as mean ± SD (**P < 0.01), 30 cells. Scale bars: 10 μm. **c** MCF-7 cells stably expressing control shRNA (shCon) or BNIP3 shRNA (shBNIP3) were treated with BBM (5 µM) for 24 h, whole-cell lysate was prepared and subjected to immunoprecipitation using anti-SNAP29, and the associated VAMP8 and BNIP3 were determined using immunoblotting. **d** MCF-7 cells co-transfected with LAMP1-mGFP, mRFP-LC3, and control shRNA (shCon) or *BNIP3* shRNA (shBNIP3) for 24 h were treated without or with BBM (5 μM) for 24 h, the colocalization of LAMP1-mGFP and mRFP-LC3 puncta was examined by confocal microscopy. The average Pearson’s correlation coefficient of LAMP1-mGFP and mRFP-LC3 colocalization was marked. Scale bars: 10 μm. **e** MCF-7 cells co-transfected with a tandem fluorescent LC3 (tfLC3) and control shRNA (shCon) or *BNIP3* shRNA (shBNIP3) were treated with BBM (5 μM) for 24 h, the colocalization of mRFP and EGFP-LC3 puncta was examined by confocal microscopy. Scale bars: 10 μm. **f** MCF-7 cells stably expressing control shRNA (shCon) or BNIP3 shRNA (shBNIP3) were treated with BBM (5 µM) for 24 h, the mitochondrial fractions were prepared, and then the LC3B-II and BNIP3 in mitochondrial fractions (Mito) were determined by western blot. The COXIV was used as a loading control

To further confirm the functional role of BNIP3 in BBM-mediated blockade of autophagosome-lysosome fusion, a genetic approach utilizing BNIP3 shRNA was employed. Depletion of BNIP3 with shRNA attenuated BBM-mediated accumulation of LC3B-II compared to that in control shRNA (shCon) cells. Since the inhibition of interaction between SNAP29 and VAMP8 was involved in BBM-mediated blockage of autophagosome-lysosome fusion, we next examined the effects of BNIP3 depletion on the colocalization of SNAP29 and VAMP8 by using immunofluorescence analysis. As shown in Fig. 6b, a mild reduction in the colocalization of SNAP29 and VAMP8 was observed in shCon cells treated with BBM, whereas the significant increase in the colocalization of SNAP29 and VAMP8 was observed in shBNIP3 cells treated with BBM. By immunoprecipitation analysis, we found that VAMP8 was co-precipitated with SNAP29 in shBNIP3 cells treated with BBM (Fig. 6c).

We also examined the effects of BNIP3 depletion on the colocalization of mRFP-LC3 and LAMP1-mGFP and the autophagic flux inhibited by BBM. The separation of mRFP-LC3 and LAMP1-mGFP was observed in shCon cells treated with BBM. In contrast, the obvious colocalization of mRFP-LC3 and LAMP1-mGFP was observed in shBNIP3 cells treated with BBM (Fig. 6d). Treatment of shCon cells with BBM caused pronounced formation of LC3 puncta that displayed both green and red fluorescence intensity producing a yellow overlay. In contrast, treatment of shBNIP3 cells with BBM led to the production of large amounts of red-only puncta (Fig. 6e). To determine the role of BNIP3 in the regulation of mitophagy mediated by BBM, the expression of LC3B-II in mitochondrial in shBNIP3 cells was determined by immunoblotting. As shown in Fig. 6f, depletion of BNIP3 with shRNA did not affect the accumulation of LC3B-II in mitochondrial induced by BBM. Taken together, these findings indicate that BNIP3 depletion abrogates BBM-mediated blockade of autophagic flux and autophagosome-lysosome fusion through recovering the interaction between SNAP29 and VAMP8.

### BNIP3 overexpression blocks autophagosome-lysosome fusion through inhibition of the interaction between SNAP29 and VAMP8

**Fig. 7:**
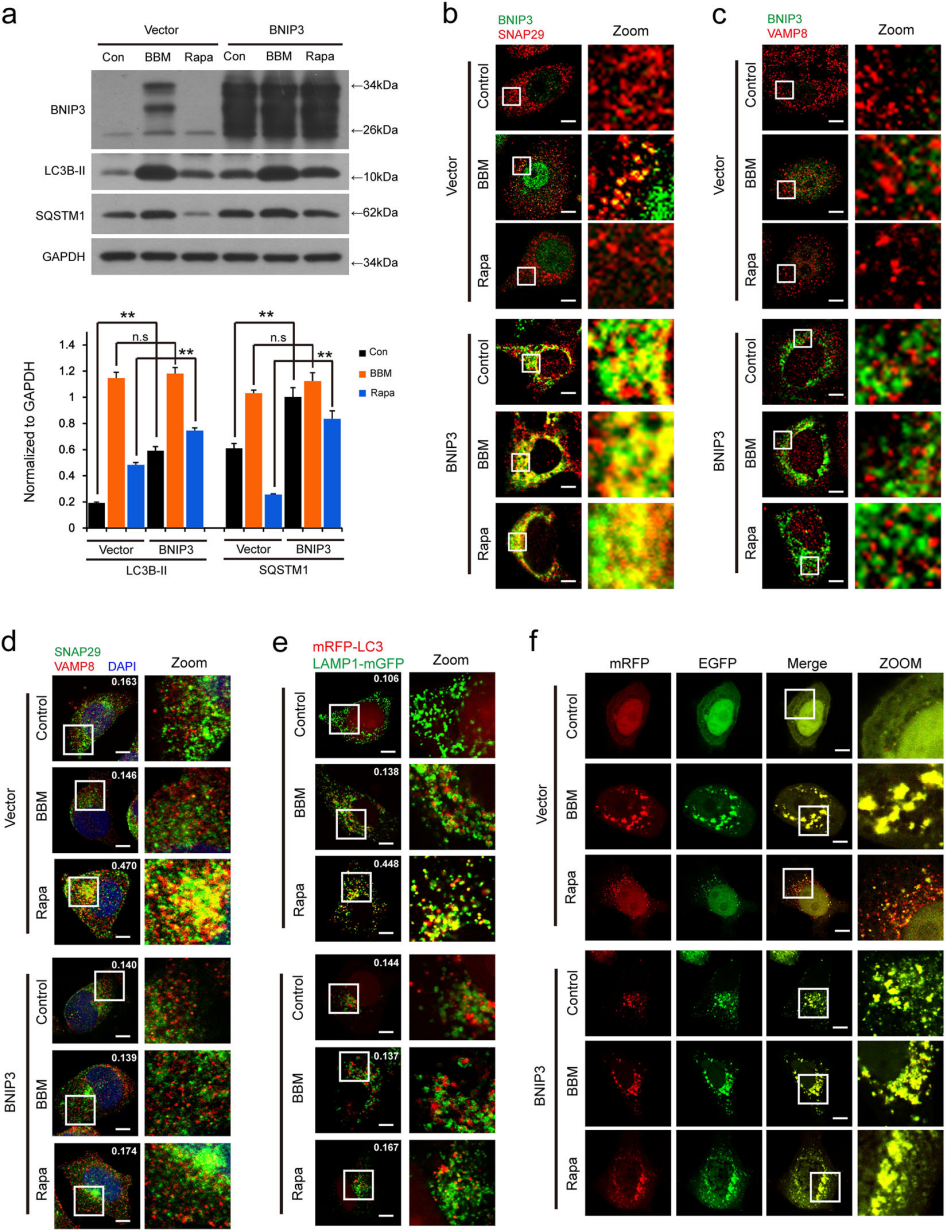
BNIP3 overexpression blocks autophagosome-lysosome fusion through inhibition of the interaction between SNAP29 and VAMP8. MCF-7 cells were transfected with control plasmid (vector) or *BNIP3* plasmid (BNIP3) for 24 h, and then treated with BBM (5 μM) and Rapa (0.25 μM) for additional 24 h. **a** The expression of BNIP3, LC3B-II, and SQSTM1 was determined by western blot. Comparison of the intensities were statistically estimated and represented as mean ± SD for three independent experiments (ns, not significant; **P < 0.01). **b** After immunostaining with BNIP3 (green) and SNAP29 (red), the colocalization of BNIP3 and SNAP29 was examined by confocal microscopy. Scale bars: 10 μm. **c** After immunostaining with BNIP3 (green) and VAMP8 (red), the colocalization of BNIP3 and VAMP8 was examined by confocal microscopy. Scale bars: 10 μm. **d** After immunostaining with SNAP29 (green) and VAMP8 (red), the colocalization of SNAP29 and VAMP8 was examined by confocal microscopy. And the average Pearson’s correlation coefficient of SNAP29 and VAMP8 colocalization was marked. Scale bars: 10 μm. **e** MCF-7 cells were co-transfected with mRFP-LC3, LAMP1-mGFP, and control plasmid (vector) or *BNIP3* plasmid (BNIP3) for 24 h, and then treated with BBM (5 μM) or Rapa (0.25 μM) for additional 24 h. The colocalization of LAMP1-mGFP and mRFP-LC3 puncta was examined by confocal microscopy. The average Pearson’s correlation coefficient of mRFP-LC3 and LAMP1-mGFP colocalization was marked. Scale bars: 10 μm. **f** MCF-7 cells were co-transfected with a tandem fluorescent LC3 (tfLC3) and control plasmid (vector) or *BNIP3* plasmid (BNIP3) for 24 h, and then treated with BBM (5 μM) or Rapa (0.25 μM) for 24 h. The colocalization of mRFP and EGFP-LC3 puncta was examined by confocal microscopy. Scale bars: 10 μm

To further assess the functional significance of BNIP3 in BBM-mediated inhibition of autophagic flux, a plasmid construct encoding BNIP3 was employed. Transfection of MCF-7 cells with BNIP3 resulted in a marked increase in levels of BNIP3 (Fig. 7a). The levels of LC3B-II and SQSTM1 were significantly elevated in BNIP3-overexpressing cells compared with that in vector control cells (Fig. 7a). And BNIP3 overexpression enhanced the LC3B-II increase and reversed the SQSTM1 decrease mediated by Rapa, but did not enhanced the LC3B-II and SQSTM1 increase mediated by BBM (Fig. 7a).

To further evaluate the functional significance of BNIP3 in BBM-mediated blockade of autophagosome-lysosome fusion, immunofluorescence analysis was employed. First, we examined the effects of BNIP3 overexpression on the colocalization of BNIP3 with SNAP29 or VAMP8. As shown in Fig. 7b, c, BNIP3 overexpression markedly enhanced the colocalization of BNIP3 with SNAP29 but not VAMP8 in response to BBM treatment.

Then we examined the effects of BNIP3 overexpression on the colocalization of SNAP29 and VAMP8 inhibited by BBM, and found that the colocalization of SNAP29 and VAMP8 was decreased by BBM treatment, but increased by Rapa treatment in vector control cells (Fig. 7d). However, BNIP3 overexpression did not potentiate the inhibition of the colocalization between SNAP29 and VAMP8 mediated by BBM, but obviously blocked the colocalization between SNAP29 and VAMP8 mediated by Rapa (Fig. 7d).

We also examined the effects of BNIP3 overexpression on the colocalization of mRFP-LC3 and LAMP1-mGFP inhibited by BBM, and found that BNIP3 overexpression did not potentiate the inhibition of the colocalization between mRFP-LC3 and LAMP1-GFP mediated by BBM, but markedly blocked the colocalization between mRFP-LC3 and LAMP1-mGFP mediated by Rapa (Fig. 7e). Finally, we examined the effects of BNIP3 overexpression on autophagic flux inhibited by BBM. BNIP3 overexpression did not potentiate BBM-mediated formation of LC3 puncta that displayed both green and red fluorescence intensity producing a yellow overlay (Fig. 7f). Interestingly, treatment of vector control cells with Rapa led to the production of large amounts of red-only puncta, whereas treatment of BNIP3-overexpressing cells with Rapa caused pronounced formation of LC3 puncta that displayed both green and red fluorescence intensity producing a yellow overlay (Fig. 7f). Taken together, these findings indicate that BNIP3 overexpression blocks autophagosome-lysosome fusion through inhibition of the interaction between SNAP29 and VAMP8.

### **SNAP29 overexpression abrogates BBM-mediated blockade of autophagosome-lysosome fusion**

**Fig. 8:**
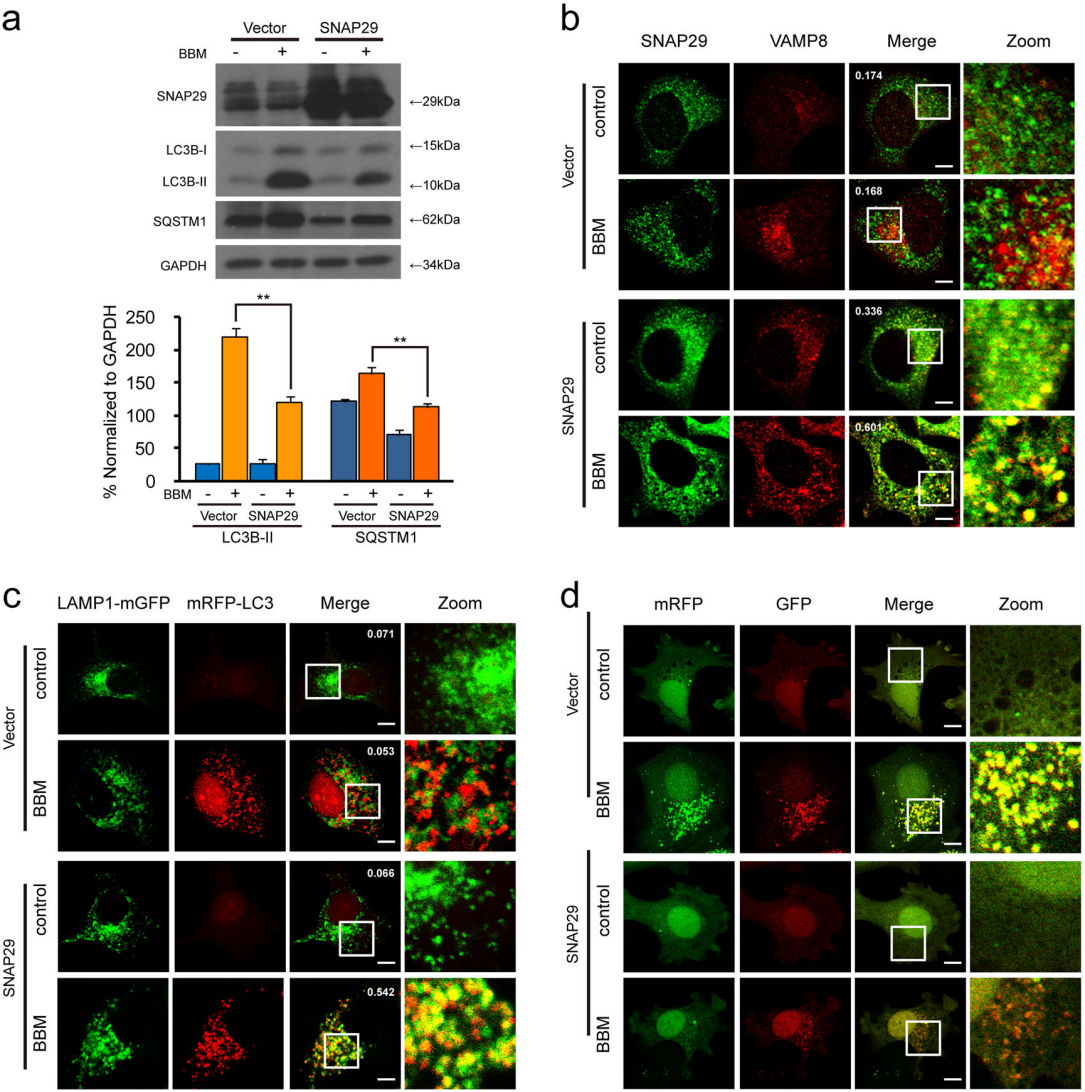
SNAP29 overexpression attenuates BBM-mediated blockade of autophagosome-lysosome fusion. MCF-7 cells were transfected with control plasmid (vector) or *SNAP29* plasmid (BNIP3) for 24 h, and then treated with BBM (5 μM) for 24 h. **a** The expression of SANP29, LC3B-II, and SQSTM1 was determined by western blot. Comparison of the intensities was statistically estimated and represented as mean ± SD for three independent experiments (ns, not significant; **P < 0.01). **b** After immunostaining with SNAP29 (green) and VAMP8 (red), the colocalization of SNAP29 and VAMP8 was examined by confocal microscopy. And the average Pearson’s correlation coefficient of SNAP29 and VAMP8 colocalization was marked. Scale bars: 10 μm. **c** MCF-7 cells were co-transfected with mRFP-LC3, LAMP1-mGFP, and control plasmid (vector) or *BNIP3* plasmid (BNIP3) for 24 h, and then treated with BBM (5 μM) for 24 h. The colocalization of LAMP1-mGFP and mRFP-LC3 puncta was examined by confocal microscopy. The average Pearson’s correlation coefficient of LAMP1-mGFP and mRFP-LC3 colocalization was marked. Scale bars: 10 μm. **d** MCF-7 cells were co-transfected with a tandem fluorescent LC3 (tfLC3) and control plasmid (vector) or *BNIP3* plasmid (BNIP3) for 24 h, and then treated with BBM (5 μM) for 24 h. The colocalization of mRFP and EGFP-LC3 puncta was examined by confocal microscopy. Scale bars: 10 μm

Our results indicated that BBM-induced BNIP3 upregulation could inhibit the interaction between SNAP29 and VAMP8, leading, in turn, to blockade of autophagosome-lysosome fusion. We speculate that overexpression of SNAP29 could reverse blockade of autophagosome-lysosome fusion mediated by BBM. To test this possibility, a plasmid construct overexpressing SNAP29 was employed. Transfection of MCF-7 cells with SNAP29 overexpression plasmid resulted in a marked increase in levels of SNAP29 (Fig. 8a). The levels of LC3B-II and SQSTM1 were significantly decreased in SNAP29-overexpressing cells compared with that in vector control cells treated with BBM (Fig. 8a).

We next examined the effects of SNAP29 overexpression on the colocalization of SNAP29 and VAMP8 by using immunofluorescence analysis. As shown in Fig. 8b, the colocalization of SNAP29 and VAMP8 could not be observed in vector control cells treated with BBM, whereas the obvious colocalization of SNAP29 and VAMP8 was observed in SNAP29-overexpressing cells treated with BBM. We also examined the effects of SNAP29 overexpression on the colocalization of mRFP-LC3 and LAMP1-mGFP and the autophagic flux inhibited by BBM. The colocalization of mRFP-LC3 and LAMP1-mGFP was not observed in vector control cells treated with BBM. In contrast, the obvious colocalization of mRFP-LC3 and LAMP1-mGFP was observed in SNAP29-overexpressing cells treated with BBM (Figure 8c). Treatment of vector control cells with BBM caused pronounced formation of LC3 puncta that displayed both green and red fluorescence intensity producing a yellow overlay. In contrast, treatment of SNAP29-overexpressing cells with BBM led to the production of large amounts of red-only puncta (Fig. 8d). Taken together, these findings suggest that overexpression of SNAP29 could compensate for blockade of autophagosome-lysosome fusion mediated by BBM treatment.

## Discussion

In the present study, we provide definitive evidence that BBM induced the accumulation of autophagosomes by inhibiting autophagosomal-lysosomal fusion. A number of evidence revealed that autophagy can be inhibited through targeting the different stage of the autophagic process^25^. The inhibitors that act at the early stage of autophagy like 3-MA and LY294002 inhibit the class III phosphatidylinositol 3-kinase and block the formation of autophagosomes^26^. At a late stage, autophagy can be inhibited by preventing the fusion of autophagosomes with lysosomes or the degradation capacity of autolysosomes^27^. Different autophagy inhibitors (e.g., Baf_,_ CQ, vinblastine, vacuolin-1, and liensinine) inhibit autophagosome-lysosome fusion through diverse mechanisms^28–32^. The mechanism of BBM-mediated blockade of autophagosome-lysosome fusion was different from that of these autophagy inhibitors. First, BBM treatment did not affect lysosomal pH; and second, BBM-mediated blockade of autophagosome-lysosome fusion was not due to reduced expression of LAMP1 and LAMP2. Interestingly, BBM blocks autophagosome-lysosome fusion by inhibiting the interaction of SNAP29 and VAMP8.

SNAREs are the critical molecular mediators of all membrane fusion events. These proteins regulate the cargo transport, secretion, autophagy, and organelle biogenesis in all cells^33,34^. It has recently been reported that autophagosomal SNARE STX17 interacting with SNAP29 (Qbc-SNARE) and the lysosomal SNARE VAMP8 is essential for autophagosome-lysosome fusion^4,35^. Depletion of any one of these proteins causes accumulation of undigested autophagosomes through blockage of autophagosome-lysosome fusion^36^. In this study, we found that BBM treatment increased the expressions of the lysosomal SNARE VAMP8, but did not affect the expression of STX17 and SNAP29. We revealed that the mechanism of BBM-blocked autophagosome-lysosome fusion was not due to reduced expression of those SNAREs, but due to block the interaction and colocalization between SNAP29 and VAMP8.

In this study, we also found that BBM treatment induced mitophagy and upregulation of BNIP3. However, the BNIP3 depletion did not affect the mitophagy induced by BBM (Fig. 6f), suggesting that BNIP3 may not be involved in the induction of mitophagy by BBM. We speculate that BNIP3 may have a critical role in blockade of autophagosome-lysosome fusion. It has recently been reported that BNIP3 expression induced autophagosome accumulation with lysosome consumption^10^. Similar to this report, our results demonstrate that BBM-mediated upregulation of BNIP3 is involved in autophagosome accumulation through blockade of autophagosome-lysosome fusion based on the following evidence. First, BBM treatment induced the expression of BNIP3 and autophagosome accumulation, and inhibited autophagosome-lysosome fusion through blockade of the interaction between SNAP29 and VAMP8. Second, BNIP3 depletion abrogated BBM-mediated blockade of autophagic flux and autophagosome-lysosome fusion through recovering the interaction between SNAP29 and VAMP8. Third, BNIP3 overexpression blocked autophagosome-lysosome fusion through inhibition of the interaction between SNAP29 and VAMP8. Fourth, BBM led to interaction and colocalization between BNIP3 and SNAP29. Finally, SNAP29 overexpression abrogated BBM-mediated blockade of autophagic flux and autophagosome-lysosome fusion. These results indicated that BBM inhibits the interaction between SNAP29 and VAMP8 through the interaction between BNIP3 and SNAP29, leading to inhibition of autophagosome-lysosome fusion.

**Fig. 9:**
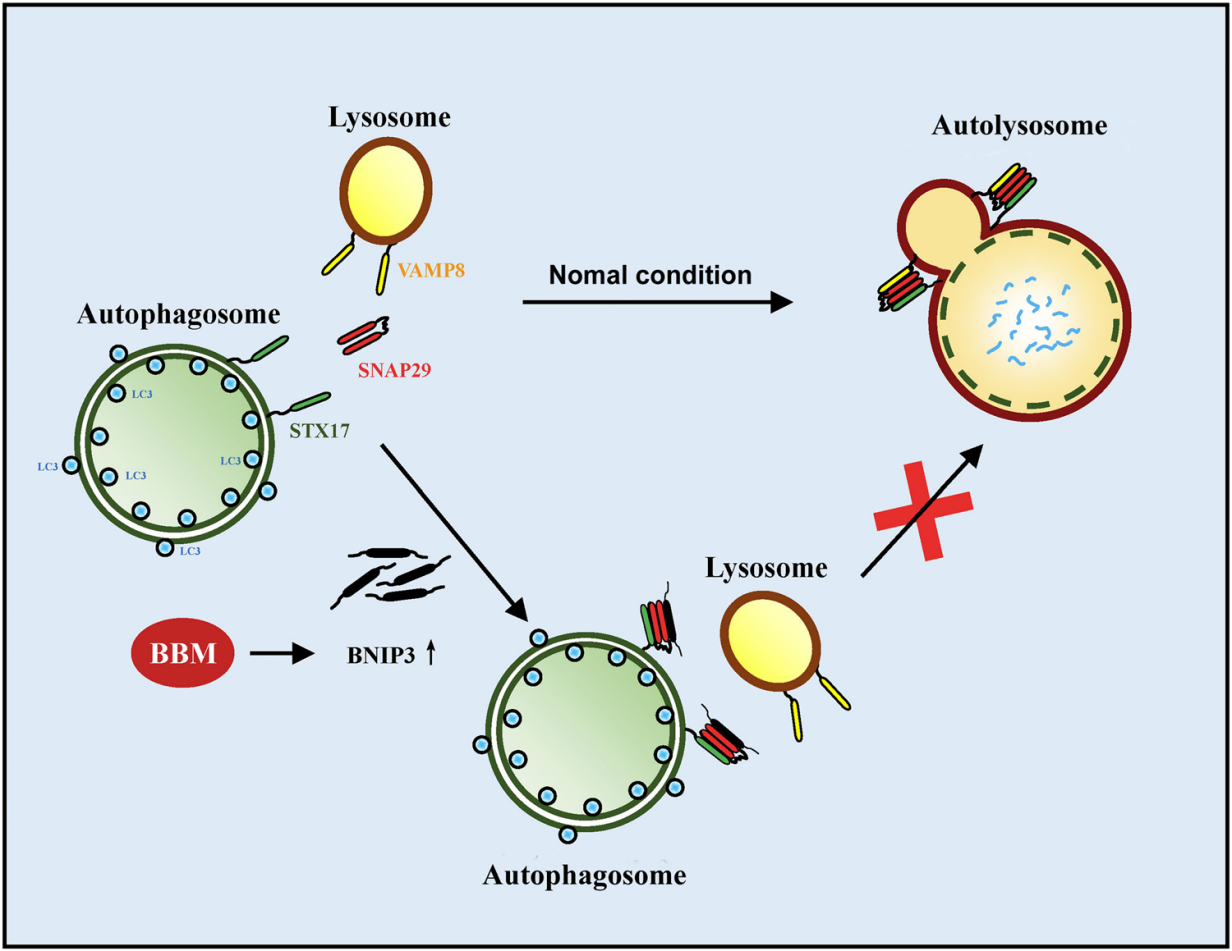
The proposed mechanism of BBM-mediated blockade of autophagosome-lysosome fusion. Under normal conditions, SNAP29 interacts with STX17 and VAMP8, which drives autophagosome-lysosome fusion. While upregulation of BNIP3 mediated by BBM, can interacts with SNAP29, which prevents the interaction between SNAP29 and VAMP8, thus blocks autophagosome-lysosome fusion

In conclusion, the present study shed new light on BBM-induced accumulation of autophagosome through inhibiting autophagosome-lysosome fusion. We provide the first evidence that BBM induces upregulation of BNIP3, which interacts with SNAP29, resulting in inhibition of the interaction between SNAP29 and VAMP8, leading, in turn, to blockade of autophagosome-lysosome fusion, and culminating in accumulation of autophagosome (Fig. 9). Our findings identify the critical role of BNIP3 in blockade of autophagosome-lysosome fusion mediated by BBM, and suggest that BBM could potentially be further developed as a novel autophagy inhibitor, which could enhance the effect of chemotherapy to cancer.

## Materials and Methods

### Antibodies and reagents

Primary antibodies used in this study were listed as following: ATG5 (12994S); ATG7 (8558); LAMP1 (9091); p-ULK1 (Ser757, 6888); and SQSTM1/P62 (5114) were purchased from Cell Signaling Technology (Boston, MA, USA). BNIP3 (sc-56167), LAMP1 (sc-20011), LAMP2 (sc-18822), SNAP29 (sc-19370-R), VAMP8 (sc-166820), and VAMP8 (sc-13060) were from Santa Cruz Biotechnology (Dallas, TX, USA). BECLIN1 (B6186), LC3B (L7543), and STX17 (HPA001204) were purchased from Sigma Aldrich (Sigma, St. Louis, MO, USA). GAPDH (AG019) was purchased from Beyotime Biotechnology (Shanghai, China). Second antibodies used in this study were listed as following: Alexa Fluor 488 goat anti-mouse (A11001); and Alexa Fluor 647 donkey anti-rabbit (A31573). These were purchased from Molecular Probes (OR, USA). Peroxidase-labeled antibody to mouse IgG (074-1802) and peroxidase-labeled antibody to rabbit IgG(074-1516) were purchased from Kirkegaard and Perry Laboratories (Atlanta, GA, USA). Reagents used in this study were follows: 4′,6-diamidino-2-phenylindole (C1006; Beyotime), Baf (11038; Cayman Chemical, Ann Arbor, MI, USA), BBM dihydrochloride (BBM, 547190; Sigma), CQ diphosphate salt (CQ, C6628; Sigma), LysoTracker^®^ Red DND-99 (L7528; Molecular Probes), Rapa (S1039; Selleck Chemicals, Houston, TX, USA), Triton X-100 (A110694; Sangon Biotech, Shanghai, China), and Tween-20 (A100777; Sangon Biotech).

### **Cell culture**

MCF-7, MDA-MB-231, and A549 cells were obtained from the American Type Culture Collection (Manassas, USA) and cultured in Dulbecco’s modified Eagle medium (Gibco, Grand Island, NY, USA) supplemented with 10% fetal bovine serum (FBS; Gibco) in 5% CO_2_ at 37 °C. Eca109 and SMMC-7721 cells were obtained from the Bena Culture Collection (Beijing, China) and cultured in RMPI 1640 (Gibco) with 10% FBS.

### Plasmids and establishment of stable cell lines

mRFP-LC3 (21075), tfLC3 (21074), and LAMP1-mGFP(34831) were obtained from Addgene (Cambridge, MA, USA). EGFP-LC3, BNIP3, and SNAP29 plasmids were constructed by Gene Chem Co. Ltd (Shanghai, China). Cells were transfected with plasmids using Lipofectamine 3000 Transfection Reagent (Invitrogen, Carlsbad, CA, USA) according to the manufacturer’s protocol. After 24 h incubation, the transfection mixture was removed and replaced with fresh complete medium. The target sequence of BNIP3 shRNA (5′-ACTGCACTTCAGCAATAAT-3′) was purchased Gene Chem Co. Ltd. To establish BNIP3 knockdown stable cell lines, 293FT cells were co-transfected with lentiviral packaging vectors pLP1, pLP2, and pLP/VSVG (Invitrogen, K4975) along with shBNIP3 or shCon plasmid using Lipofectamine 3000 (Invitrogen, L3000015) according to the manufacturer’s protocols. Forty-eight hours later, supernatant containing the lentivirus was harvested and infected with MCF-7 cells. Cells were subsequently selected with 4 μg/mL puromycin (Sigma, P9620) to establish stable cell lines.

### Western blots and immunoprecipitation

For western blot, cells were treated and lysed in cell lysis buffer (Beyotime Biotechnology) to obtain protein lysate and the concentration measured using an enhanced BCA protein assay kit (Beyotime Biotechnology), then cell lysate was loaded onto SDS-polyacrylamide gel electrophoresis (SDS-PAGE) gels and transferred to polyvinylidene difluoride membranes (Bio-Rad, Hercules, CA, USA), after blocking with 5% fat-free dry milk in Tris-buffered saline (10 mM Tris-base and 150 mM NaCl, pH 7.6), containing 0.1% Tween-20, membranes were incubated with primary antibodies. Horseradish peroxidase-labeled secondary antibodies were used to detect primary antibodies. Bands were visualized with Clarity Western ECL Substrate (Bio-Rad). For immunoprecipitation, total protein lysates were obtained as described; equal quantities of proteins were incubated with primary antibodies at 4 °C on a rocking platform. Immune complexes were collected with protein A/G agarose beads (Beyotime Technology) followed by five times wash in phosphate-buffered saline (PBS), samples were subjected to SDS-PAGE and western blot. Quantification relative to GAPDH by densitometric analysis using the Quality One software (Bio-Rad).

### Immunofluorescence

Cells were seeded on coverslips and cultured in 24-well plates for 24 h, and then transfected with respective plasmids. After transfection for 24 h, cells were treated with drugs for the indicated time. For different experimental conditions, lysosomes and mitochondria were stained with LysoTracker Red DND-99 (Molecular Probes, Carlsbad, USA) and MitoTracker Deep Red FM (Molecular Probes, Carlsbad, USA), respectively, according to the manufacturer’s instructions. Cells were fixed with 4% formaldehyde (Beyotime Biotechnology) for 30 min, permeabilized with 0.1% Triton X-100 in PBS for 5 min, blocked with 10% goat serum (Beyotime Biotechnology) in PBS for 30 min. Cells were incubated with various primary antibodies at 4 °C overnight, followed by the appropriate secondary antibodies Alexa Fluor 488 goat anti-mouse (Molecular Probes, Waltham, MA, USA) or Alexa Fluor 647 donkey anti-rabbit (Molecular Probes), at 37°C for 1 h. Cells were viewed using a laser-scanning confocal microscope (Zeiss, Germany). All images were analyzed by ImageJ software (MD, USA)

### RNA extraction and quantitative reverse-transcription-PCR

Cells were seeded in six-well plates and cultured. Total RNAs were extracted from cultured cells using TRIzol reagent (Invitrogen) according to the manufacturer’s protocol. Gene expression was verified by using AZpolarisTM Cdna Synthesis Kit (Azanno Biotech, Gothenburg, Sweden) for reverse-transcription and RealMaster Mix (Tiangen Biotech, Beijing, China) for reverse-transcription-PCR, according to the manufacturer’s instructions. The primers used were as follows: BNIP3-FWD (5′-CAGGGCTCCTGGGTAGAACT-3′), BNIP3-REV (5′-CTACTCCGTCCAGACTCATGC-3′); GAPDH-FWD (5′-TTGGTATCGTGGAAGGACTCA-3′) and GAPDH-REV (5′-TGTCATCATATTTGGCAGGTT-3′).

### Statistical analysis

Statistical analysis was performed with SPSS 17.0 software (Chicago, IL, USA). Some data were normalized to vehicle or scrambled control and analyzed with two-tailed Student’s *T*-tests. ns, not significant; 0.01 ≤ *P < 0.05; **P<0.01.
